# Estimating the COVID-19 mortality burden over two full years of the pandemic in Malaysia

**DOI:** 10.1016/j.lanwpc.2022.100456

**Published:** 2022-04-27

**Authors:** LeeAnn Tan, Shubash Shander Ganapathy, Yee Mang Chan, Nazirah Alias, Nur Hamizah Nasaruddin, Wan-Fei Khaw, Azahadi Omar

**Affiliations:** a Institute for Public Health, National Institutes of Health, Ministry of Health, Selangor, Malaysia; b Sector for Biostatistics and Data Repository, National Institutes of Health, Ministry of Health, Selangor, Malaysia

Malaysia reported its first known local transmission of the severe acute respiratory syndrome coronavirus 2 (SARS-CoV-2) on 5 February 2020.^1^ On 17 March 2020, the first two deaths from coronavirus disease 2019 (COVID-19) in the country occurred—just a week after the World Health Organization (WHO) formally declared the growing outbreak a pandemic.^2^ Two years into the current pandemic, there have been over 32,000 deaths linked to COVID-19 in Malaysia over three major epidemic waves and a cumulative case count of over three million, making it one of the most affected countries in the Western Pacific region.^3^ Absolute death counts are often the metric of choice in official reports (and in accompanying graphics—often in large, bold numbers—circulated through social media or chat groups to be fixated upon by the populace) summarising the latest COVID-19 statistics. Burden of disease approaches, such as measuring years of life lost (YLL) owing to premature death from a disease, offer a more appropriate metric than number of deaths for measuring the mortality burden of a disease on a population and sub-populations by recognising that deaths at younger ages have a greater impact on population health. This study aims to estimate the burden of mortality from COVID-19 by calculating YLL to COVID-19 over two full years of the pandemic in Malaysia, to compare YLL rates across different states, and to compare the impact of mortality directly attributable to COVID-19 relative to deaths from other leading causes of disease and injury in the country in pre-pandemic times.

Data on individual COVID-19 deaths that occurred up to 5 February 2022 (which marks the two-year anniversary of the country's first-known locally transmitted COVID-19 case) were obtained from a public repository provided and updated regularly by the Ministry of Health Malaysia.^4^ To calculate YLL directly attributable to COVID-19 for each age/sex group, we multiplied the number of deaths in each group by the age- and sex-conditional life expectancy for the same group as defined in the national life table published by the Department of Statistics Malaysia (DOSM).^5^ We used the national life table for the year 2017^6^—the same one employed in the most recently available national burden of disease estimates.^7^ To facilitate international comparison, we also present results separately for calendar years 2020 and 2021, and additionally calculated YLL applying life expectancy values from the Global Burden of Disease Study 2019 (GBD 2019) reference life table^8^ (see supplementary material). To calculate YLL rates, national and state-level population data were obtained from mid-year population estimates for the year 2021, also published by DOSM.^9^

Two years after the first cases of COVID-19 surfaced in Malaysia, there have been 32,063 deaths recorded in the country. Most deaths (31,059 out of 32,063 deaths) occurred in 2021, and the highest number of deaths occurred in the 60-64 years age group (13%) in men and in the 80+ years age group (18%) in women (Supplementary Table 1a). Male deaths outnumbered female deaths by a ratio of 1·35 to 1.

We estimated that COVID-19 accounted for 683,903 YLL over the past two years—approximately 21 years lost per person who died of COVID-19, and 1,998 years per 100,000 people. Men lost a greater amount of YLL per 100,000 people compared to women (2,132 vs. 1,853). YLL were highest in the 55–59 years age group in men, and in the 45-49 years age group in women (Figure 1). The state of Selangor lost the greatest amount of YLL per capita, with the Federal Territory of Labuan coming in a close second at 3,473 years and 3,457 years per 100,000 people, respectively (Figure 2; Supplementary Table 3). The mortality burden directly attributable to COVID-19 over the 2021 calendar year (665,029 YLL) exceeds that caused by ischaemic heart disease, which was the leading cause of fatal burden in the country in pre-pandemic times (Figure 3). COVID-19 also accounted for more than two times the amount of YLL associated with lower respiratory tract infections in 2017 (Figure 3).^7^

**Figure 1:**
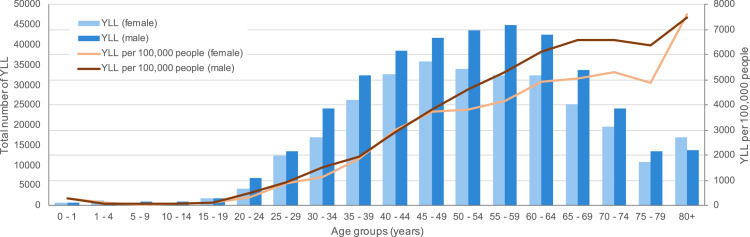
Estimated years of life lost (YLL) due to COVID-19 by age and sex over two full years of the pandemic in Malaysia starting from 5 February 2020.

**Figure 2:**
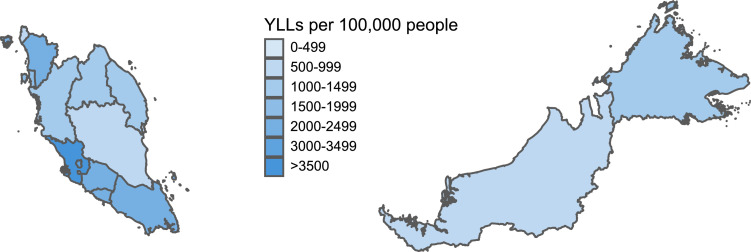
Estimated years of life lost (YLL) per 100,000 people by individual states and federal territories, Malaysia.

**Figure 3:**
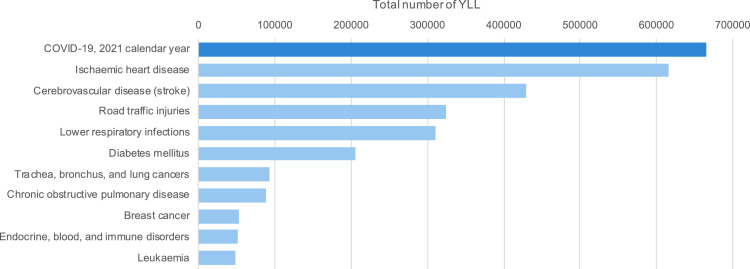
Comparison of overall estimated years of life lost (YLL) due to COVID-19 in calendar year 2021 with YLL due to the 10 leading causes of fatal burden in Malaysia in 2017^7^.

It is notable that the mortality burden alone of COVID-19 approximates that of the total burden of disease (conventionally represented by the summary measure Disability-Adjusted Life Years or DALYs, which also takes into account Years Lived with Disability [YLD] in addition to YLL) of ischaemic heart disease—the leading cause of disease in 2017 (665,029 vs 688,594).^7^ A limitation of our brief report is the absence of data on YLD contributed by non-fatal cases, although evidence from other countries indicates that most of the burden due to COVID-19 arose from fatal cases with YLL comprising 95 to 99·3% of the total disease burden.^10^, ^11^, ^12^, ^13^ Future work is underway to quantify the morbidity burden and other effects of COVID-19, including “long COVID”—a collective term denoting prolonged and persistent symptoms in those who have recovered from SARS-CoV-2 infection^14^—which will require further understanding in order to assign appropriate disability weights for the calculation of YLD. Competing risk due to death from other causes should also be considered when interpreting these estimates, where it is likely that at least some of the deaths attributed to COVID-19 have replaced deaths that would have occurred due to other causes.^15^

These YLL estimates highlight that the COVID-19 pandemic has caused a considerable mortality impact on the Malaysian population. While we recognise that these preliminary estimates do not even begin to quantify the magnitude of losses experienced by other wider ranging effects of the pandemic beyond loss of life, we hope that reporting the comparative population health impact of COVID-19 lends perspective to the debate on whether the implementation of radical mitigation measures including multiple movement control orders (i.e., lockdowns) were necessary. Continued estimation of the disease burden associated with COVID-19 at regular intervals will be helpful in quantifying potential losses averted by the rollout of the National COVID-19 Immunisation Programme (*Program Imunisasi COVID-19 Kebangsaan*, PICK).

## Funding

This work did not receive any specific grant from funding agencies in the public, commercial, or not-for-profit sectors.

## Contributors

LT and SSG conceptualised the study and initial structure of the brief report. LT, YMC, NA, NHN, and WFK contributed towards data curation, analysis, validation and interpretation of results. SSG and AO supervised the study. LT wrote the original draft and all co-authors worked together to revise it. LT wrote the final version; all co-authors reviewed and approved it.

## Availability of data and materials

Data used in this study are publicly available on GitHub at https://github.com/MoH-Malaysia/covid19-public/)

## Declaration of interests

We declare no competing interests.

## Appendix Supplementary materials

Image, application 1
