# Supplementary material for: Estimating the COVID-19 mortality burden over two full years of the pandemic in Malaysia
Source: Lancet Reg Health West Pac. 2022 Apr 27;22:100456. doi: 10.1016/j.lanwpc.2022.100456 (PMC9042593; doi:10.1016/j.lanwpc.2022.100456)
Supplement: Supplementary file 1 [file mmc1.docx]

**Supplementary materials**

| Age group | Male | | | | |  | Female | | | | |
| --- | --- | --- | --- | --- | --- | --- | --- | --- | --- | --- | --- |
|  | Population | LE | Deaths (%) | YLL (%) | YLL per  100,000 people |  | Population | LE | Deaths (%) | YLL (%) | YLL per  100,000 people |
| <1 | 280700 | 72·7 | 11 (0·06) | 799·7 (0·21) | 284·9 |  | 265440 | 77·4 | 10 (0·07) | 774 (0·25) | 291·6 |
| 1 - 4 | 1122800 | 72·1 | 11 (0·06) | 793·1 (0·21) | 70·6 |  | 1061760 | 76·8 | 15 (0·11) | 1152 (0·38) | 108·5 |
| 5 - 9 | 1391200 | 68·2 | 16 (0·09) | 1091·2 (0·29) | 78·4 |  | 1313400 | 72·9 | 8 (0·06) | 583·2 (0·19) | 44·4 |
| 10 - 14 | 1290300 | 63·3 | 15 (0·08) | 949·5 (0·25) | 73·6 |  | 1224800 | 68·0 | 11 (0·08) | 748 (0·25) | 61·1 |
| 15 - 19 | 1390800 | 58·4 | 29 (0·16) | 1693·6 (0·45) | 121·8 |  | 1308900 | 63·1 | 28 (0·21) | 1766·8 (0·58) | 135·0 |
| 20 - 24 | 1420100 | 53·7 | 127 (0·69) | 6819·9 (1·8) | 480·2 |  | 1338700 | 58·2 | 74 (0·54) | 4306·8 (1·41) | 321·7 |
| 25 - 29 | 1476300 | 49·0 | 278 (1·51) | 13622 (3·6) | 922·7 |  | 1391100 | 53·3 | 232 (1·7) | 12365·6 (4·05) | 888·9 |
| 30 - 34 | 1596400 | 44·2 | 549 (2·98) | 24265·8 (6·41) | 1520·0 |  | 1460900 | 48·4 | 350 (2·56) | 16940 (5·55) | 1159·6 |
| 35 - 39 | 1643800 | 39·5 | 819 (4·45) | 32350·5 (8·54) | 1968·0 |  | 1399100 | 43·6 | 602 (4·41) | 26247·2 (8·6) | 1876·0 |
| 40 - 44 | 1313900 | 34·9 | 1103 (5·99) | 38494·7 (10·16) | 2929·8 |  | 1094300 | 38·8 | 845 (6·19) | 32786 (10·75) | 2996·1 |
| 45 - 49 | 1090300 | 30·5 | 1366 (7·42) | 41663 (11) | 3821·2 |  | 961500 | 34·2 | 1051 (7·7) | 35944·2 (11·78) | 3738·3 |
| 50 - 54 | 943700 | 26·2 | 1664 (9·04) | 43596·8 (11·51) | 4619·8 |  | 895400 | 29·6 | 1151 (8·43) | 34069·6 (11·17) | 3805·0 |
| 55 - 59 | 843400 | 22·2 | 2025 (11) | 44955 (11·87) | 5330·2 |  | 780100 | 25·2 | 1286 (9·42) | 32407·2 (10·62) | 4154·2 |
| 60 - 64 | 697700 | 18·4 | 2314 (12·57) | 42577·6 (11·24) | 6102·6 |  | 659800 | 21·0 | 1549 (11·35) | 32529 (10·66) | 4930·1 |
| 65 - 69 | 512200 | 15·0 | 2254 (12·24) | 33810 (8·92) | 6600·9 |  | 494400 | 17·1 | 1468 (10·75) | 25102·8 (8·23) | 5077·4 |
| 70 - 74 | 363900 | 11·7 | 2056 (11·17) | 24055·2 (6·35) | 6610·4 |  | 369000 | 13·4 | 1459 (10·69) | 19550·6 (6·41) | 5298·3 |
| 75 - 79 | 212400 | 8·7 | 1561 (8·48) | 13580·7 (3·58) | 6393·9 |  | 224200 | 9·9 | 1104 (8·09) | 10929·6 (3·58) | 4874·9 |
| 80+ | 183200 | 6·2 | 2215 (12·03) | 13733 (3·62) | 7496·2 |  | 221800 | 7·0 | 2407 (17·63) | 16849 (5·52) | 7596·5 |
| **Total** |  |  | **18413 (100)** | **378851·3 (100)** | **2131·6** |  |  |  | **13650 (100)** | **305051·6 (100)** | **1852·8** |
| LE, life expectancy; YLL, years of life lost | | | |  |  |  |  |  |  |  |  |
| LE values based on national life table for the year 2017 published by the Department of Statistics Malaysia (DOSM) | | | | | | | | |  |  |  |

**Supplementary Table 1.** Death counts and years of life lost (YLL) due to COVID-19 by age and sex over two years of the pandemic (up to 5 February 2022) in Malaysia; using LE values from the 2017 national life table

| Age group | Male | | | | |  | Female | | | | |
| --- | --- | --- | --- | --- | --- | --- | --- | --- | --- | --- | --- |
|  | Population | LE | Deaths (%) | YLL (%) | YLL per  100,000 people |  | Population | LE | Deaths (%) | YLL (%) | YLL per  100,000 people |
| <1 | 280700 | 72·7 | 0 (0) | 0 (0) | 0·0 |  | 265440 | 77·4 | 0 (0) | 0 (0) | 0·0 |
| 1 - 4 | 1122800 | 72·1 | 1 (0·3) | 72·1 (1·14) | 6·4 |  | 1061760 | 76·8 | 2 (1·1) | 153·6 (3·84) | 14·5 |
| 5 - 9 | 1391200 | 68·2 | 0 (0) | 0 (0) | 0·0 |  | 1313400 | 72·9 | 1 (0·55) | 72·9 (1·82) | 5·6 |
| 10 - 14 | 1290300 | 63·3 | 1 (0·3) | 63·3 (1) | 4·9 |  | 1224800 | 68·0 | 0 (0) | 0 (0) | 0·0 |
| 15 - 19 | 1390800 | 58·4 | 0 (0) | 0 (0) | 0·0 |  | 1308900 | 63·1 | 1 (0·55) | 63·1 (1·58) | 4·8 |
| 20 - 24 | 1420100 | 53·7 | 2 (0·6) | 107·4 (1·7) | 7·6 |  | 1338700 | 58·2 | 2 (1·1) | 116·4 (2·91) | 8·7 |
| 25 - 29 | 1476300 | 49·0 | 8 (2·4) | 392 (6·2) | 26·6 |  | 1391100 | 53·3 | 2 (1·1) | 106·6 (2·67) | 7·7 |
| 30 - 34 | 1596400 | 44·2 | 4 (1·2) | 176·8 (2·8) | 11·1 |  | 1460900 | 48·4 | 3 (1·65) | 145·2 (3·63) | 9·9 |
| 35 - 39 | 1643800 | 39·5 | 12 (3·59) | 474 (7·49) | 28·8 |  | 1399100 | 43·6 | 4 (2·2) | 174·4 (4·36) | 12·5 |
| 40 - 44 | 1313900 | 34·9 | 7 (2·1) | 244·3 (3·86) | 18·6 |  | 1094300 | 38·8 | 6 (3·3) | 232·8 (5·83) | 21·3 |
| 45 - 49 | 1090300 | 30·5 | 15 (4·49) | 457·5 (7·23) | 42·0 |  | 961500 | 34·2 | 12 (6·59) | 410·4 (10·27) | 42·7 |
| 50 - 54 | 943700 | 26·2 | 29 (8·68) | 759·8 (12·01) | 80·5 |  | 895400 | 29·6 | 17 (9·34) | 503·2 (12·59) | 56·2 |
| 55 - 59 | 843400 | 22·2 | 37 (11·08) | 821·4 (12·99) | 97·4 |  | 780100 | 25·2 | 15 (8·24) | 378 (9·46) | 48·5 |
| 60 - 64 | 697700 | 18·4 | 56 (16·77) | 1030·4 (16·29) | 147·7 |  | 659800 | 21·0 | 30 (16·48) | 630 (15·76) | 95·5 |
| 65 - 69 | 512200 | 15·0 | 52 (15·57) | 780 (12·33) | 152·3 |  | 494400 | 17·1 | 19 (10·44) | 324·9 (8·13) | 65·7 |
| 70 - 74 | 363900 | 11·7 | 33 (9·88) | 386·1 (6·1) | 106·1 |  | 369000 | 13·4 | 24 (13·19) | 321·6 (8·05) | 87·2 |
| 75 - 79 | 212400 | 8·7 | 33 (9·88) | 287·1 (4·54) | 135·2 |  | 224200 | 9·9 | 19 (10·44) | 188·1 (4·71) | 83·9 |
| 80+ | 183200 | 6·2 | 44 (13·17) | 272·8 (4·31) | 148·9 |  | 221800 | 7·0 | 25 (13·74) | 175 (4·38) | 78·9 |
| **Total** |  |  | **334 (100)** | **6325 (100)** | **35·6** |  |  |  | **182 (100)** | **3996·2 (100)** | **24·3** |
| LE, life expectancy; YLL, years of life lost | | | |  |  |  |  |  |  |  |  |
| LE values based on national life table for the year 2017 published by the Department of Statistics Malaysia (DOSM) | | | | | | | | |  |  |  |

**Supplementary Table 1b.** Death counts and years of life lost (YLL) due to COVID-19 by age and sex in calendar year 2020 in Malaysia; using LE values from the 2017 national life table

| Age group | Male | | | | |  | Female | | | | |
| --- | --- | --- | --- | --- | --- | --- | --- | --- | --- | --- | --- |
|  | Population | LE | Deaths (%) | YLL (%) | YLL per  100,000 people |  | Population | LE | Deaths (%) | YLL (%) | YLL per  100,000 people |
| <1 | 280700 | 72·7 | 10 (0·06) | 727 (0·2) | 259·0 |  | 265440 | 77·4 | 10 (0·08) | 774 (0·26) | 291·6 |
| 1 - 4 | 1122800 | 72·1 | 10 (0·06) | 721 (0·2) | 64·2 |  | 1061760 | 76·8 | 12 (0·09) | 921·6 (0·31) | 86·8 |
| 5 - 9 | 1391200 | 68·2 | 16 (0·09) | 1091·2 (0·3) | 78·4 |  | 1313400 | 72·9 | 7 (0·05) | 510·3 (0·17) | 38·9 |
| 10 - 14 | 1290300 | 63·3 | 14 (0·08) | 886·2 (0·24) | 68·7 |  | 1224800 | 68·0 | 10 (0·08) | 680 (0·23) | 55·5 |
| 15 - 19 | 1390800 | 58·4 | 29 (0·16) | 1693·6 (0·46) | 121·8 |  | 1308900 | 63·1 | 26 (0·2) | 1640·6 (0·55) | 125·3 |
| 20 - 24 | 1420100 | 53·7 | 125 (0·7) | 6712·5 (1·82) | 472·7 |  | 1338700 | 58·2 | 71 (0·54) | 4132·2 (1·39) | 308·7 |
| 25 - 29 | 1476300 | 49·0 | 268 (1·5) | 13132 (3·57) | 889·5 |  | 1391100 | 53·3 | 228 (1·72) | 12152·4 (4·09) | 873·6 |
| 30 - 34 | 1596400 | 44·2 | 541 (3·04) | 23912·2 (6·5) | 1497·9 |  | 1460900 | 48·4 | 343 (2·59) | 16601·2 (5·59) | 1136·4 |
| 35 - 39 | 1643800 | 39·5 | 799 (4·49) | 31560·5 (8·58) | 1920·0 |  | 1399100 | 43·6 | 596 (4·5) | 25985·6 (8·75) | 1857·3 |
| 40 - 44 | 1313900 | 34·9 | 1090 (6·12) | 38041 (10·34) | 2895·3 |  | 1094300 | 38·8 | 830 (6·26) | 32204 (10·84) | 2942·9 |
| 45 - 49 | 1090300 | 30·5 | 1332 (7·48) | 40626 (11·04) | 3726·1 |  | 961500 | 34·2 | 1027 (7·75) | 35123·4 (11·82) | 3653·0 |
| 50 - 54 | 943700 | 26·2 | 1618 (9·09) | 42391·6 (11·52) | 4492·1 |  | 895400 | 29·6 | 1122 (8·47) | 33211·2 (11·18) | 3709·1 |
| 55 - 59 | 843400 | 22·2 | 1966 (11·04) | 43645·2 (11·86) | 5174·9 |  | 780100 | 25·2 | 1256 (9·48) | 31651·2 (10·66) | 4057·3 |
| 60 - 64 | 697700 | 18·4 | 2217 (12·45) | 40792·8 (11·09) | 5846·8 |  | 659800 | 21·0 | 1493 (11·27) | 31353 (10·56) | 4751·9 |
| 65 - 69 | 512200 | 15·0 | 2179 (12·24) | 32685 (8·88) | 6381·3 |  | 494400 | 17·1 | 1425 (10·75) | 24367·5 (8·2) | 4928·7 |
| 70 - 74 | 363900 | 11·7 | 1994 (11·2) | 23329·8 (6·34) | 6411·0 |  | 369000 | 13·4 | 1413 (10·66) | 18934·2 (6·37) | 5131·2 |
| 75 - 79 | 212400 | 8·7 | 1491 (8·37) | 12971·7 (3·52) | 6107·2 |  | 224200 | 9·9 | 1075 (8·11) | 10642·5 (3·58) | 4746·9 |
| 80+ | 183200 | 6·2 | 2109 (11·84) | 13075·8 (3·55) | 7137·4 |  | 221800 | 7·0 | 2307 (17·41) | 16149 (5·44) | 7280·9 |
| **Total** |  |  | **17808 (100)** | **367995·1 (100)** | **2070·5** |  |  |  | **13251 (100)** | **297033·9 (100)** | **1804·1** |
| LE, life expectancy; YLL, years of life lost | | | |  |  |  |  |  |  |  |  |
| LE values based on national life table for the year 2017 published by the Department of Statistics Malaysia (DOSM) | | | | | | | | |  |  |  |

**Supplementary Table 1c.** Death counts and years of life lost (YLL) due to COVID-19 by age and sex in calendar year 2021 in Malaysia; using LE values from the 2017 national life table

| Age group | Male | | | | |  | Female | | | | |
| --- | --- | --- | --- | --- | --- | --- | --- | --- | --- | --- | --- |
|  | Population | LE | Deaths (%) | YLL (%) | YLL per  100,000 people |  | Population | LE | Deaths (%) | YLL (%) | YLL per  100,000 people |
| <1 | 280700 | 88·9 | 11 (0·06) | 977·6 (0·17) | 348·3 |  | 265440 | 88·9 | 10 (0·07) | 888·7 (0·21) | 334·8 |
| 1 - 4 | 1122800 | 88·0 | 11 (0·06) | 968 (0·17) | 86·2 |  | 1061760 | 88·0 | 15 (0·11) | 1320 (0·32) | 124·3 |
| 5 - 9 | 1391200 | 84·0 | 16 (0·09) | 1344·5 (0·23) | 96·6 |  | 1313400 | 84·0 | 8 (0·06) | 672·2 (0·16) | 51·2 |
| 10 - 14 | 1290300 | 79·0 | 15 (0·08) | 1185·7 (0·2) | 91·9 |  | 1224800 | 79·0 | 11 (0·08) | 869·5 (0·21) | 71·0 |
| 15 - 19 | 1390800 | 74·1 | 29 (0·16) | 2147·9 (0·37) | 154·4 |  | 1308900 | 74·1 | 28 (0·21) | 2073·9 (0·5) | 158·4 |
| 20 - 24 | 1420100 | 69·1 | 127 (0·69) | 8776·7 (1·5) | 618·0 |  | 1338700 | 69·1 | 74 (0·54) | 5114 (1·22) | 382·0 |
| 25 - 29 | 1476300 | 64·1 | 278 (1·51) | 17833·5 (3·05) | 1208·0 |  | 1391100 | 64·1 | 232 (1·7) | 14882·6 (3·55) | 1069·8 |
| 30 - 34 | 1596400 | 59·2 | 549 (2·98) | 32498·8 (5·55) | 2035·8 |  | 1460900 | 59·2 | 350 (2·56) | 20718·7 (4·95) | 1418·2 |
| 35 - 39 | 1643800 | 54·3 | 819 (4·45) | 44432·9 (7·59) | 2703·1 |  | 1399100 | 54·3 | 602 (4·41) | 32660·1 (7·8) | 2334·4 |
| 40 - 44 | 1313900 | 49·3 | 1103 (5·99) | 54397·1 (9·29) | 4140·1 |  | 1094300 | 49·3 | 845 (6·19) | 41673·2 (9·95) | 3808·2 |
| 45 - 49 | 1090300 | 44·4 | 1366 (7·42) | 60695·9 (10·37) | 5566·9 |  | 961500 | 44·4 | 1051 (7·7) | 46699·4 (11·15) | 4856·9 |
| 50 - 54 | 943700 | 39·6 | 1664 (9·04) | 65952·2 (11·26) | 6988·7 |  | 895400 | 39·6 | 1151 (8·43) | 45619·6 (10·89) | 5094·9 |
| 55 - 59 | 843400 | 34·9 | 2025 (11) | 70702·6 (12·08) | 8383·0 |  | 780100 | 34·9 | 1286 (9·42) | 44900·5 (10·72) | 5755·7 |
| 60 - 64 | 697700 | 30·3 | 2314 (12·57) | 70006·5 (11·96) | 10033·9 |  | 659800 | 30·3 | 1549 (11·35) | 46862·6 (11·19) | 7102·5 |
| 65 - 69 | 512200 | 25·7 | 2254 (12·24) | 57884·7 (9·89) | 11301·2 |  | 494400 | 25·7 | 1468 (10·75) | 37699·6 (9) | 7625·3 |
| 70 - 74 | 363900 | 21·3 | 2056 (11·17) | 43768·5 (7·48) | 12027·6 |  | 369000 | 21·3 | 1459 (10·69) | 31059·5 (7·41) | 8417·2 |
| 75 - 79 | 212400 | 17·1 | 1561 (8·48) | 26698·6 (4·56) | 12570·0 |  | 224200 | 17·1 | 1104 (8·09) | 18882·3 (4·51) | 8422·1 |
| 80 - 84 | 106951 | 13·2 | 1217 (6·61) | 16111·5 (2·75) | 15064·4 |  | 135973 | 13·2 | 1154 (8·45) | 15277·5 (3·65) | 11235·7 |
| 85 - 89 | 52819 | 10·0 | 693 (3·76) | 6923·2 (1·18) | 13107·4 |  | 63265 | 10·0 | 743 (5·44) | 7422·7 (1·77) | 11732·7 |
| 90 - 95 | 18019 | 7·6 | 237 (1·29) | 1805·4 (0·31) | 10019·5 |  | 18916 | 7·6 | 367 (2·69) | 2795·7 (0·67) | 14779·5 |
| 95+ | 5411 | 5·9 | 68 (0·37) | 402·7 (0·07) | 7442·6 |  | 3646 | 5·9 | 143 (1·05) | 846·9 (0·2) | 23227·4 |
| **Total** |  |  | **18413 (100)** | **585514·5 (100)** | **3294·4** |  |  |  | **13650 (100)** | **418939·1 (100)** | **2544·5** |
| LE, life expectancy; YLL, years of life lost | | | |  |  |  |  |  |  |  |  |
| LE values based on reference life table from GBD 2019, published by the Institute for Health Metrics and Evaluation (IHME) | | | | | | | | |  |  |  |
|  | | | | | | | | |  |  |  |

**Supplementary Table 2a.** Death counts and years of life lost (YLL) due to COVID-19 by age and sex over two years of the pandemic (up to 5 February 2022) in Malaysia; using LE values from the GBD 2019 reference life table (this table assigns the same values to both males and females)

| Age group | Male | | | | |  | Female | | | | |
| --- | --- | --- | --- | --- | --- | --- | --- | --- | --- | --- | --- |
|  | Population | LE | Deaths (%) | YLL (%) | YLL per  100,000 people |  | Population | LE | Deaths (%) | YLL (%) | YLL per  100,000 people |
| <1 | 280700 | 88·9 | 0 (0) | 0 (0) | 0·0 |  | 265440 | 88·9 | 0 (0) | 0 (0) | 0·0 |
| 1 - 4 | 1122800 | 88·0 | 1 (0·3) | 88 (0·88) | 7·8 |  | 1061760 | 88·0 | 2 (1·1) | 176 (3·19) | 16·6 |
| 5 - 9 | 1391200 | 84·0 | 0 (0) | 0 (0) | 0·0 |  | 1313400 | 84·0 | 1 (0·55) | 84 (1·52) | 6·4 |
| 10 - 14 | 1290300 | 79·0 | 1 (0·3) | 79 (0·79) | 6·1 |  | 1224800 | 79·0 | 0 (0) | 0 (0) | 0·0 |
| 15 - 19 | 1390800 | 74·1 | 0 (0) | 0 (0) | 0·0 |  | 1308900 | 74·1 | 1 (0·55) | 74·1 (1·34) | 5·7 |
| 20 - 24 | 1420100 | 69·1 | 2 (0·6) | 138·2 (1·39) | 9·7 |  | 1338700 | 69·1 | 2 (1·1) | 138·2 (2·51) | 10·3 |
| 25 - 29 | 1476300 | 64·1 | 8 (2·4) | 513·2 (5·16) | 34·8 |  | 1391100 | 64·1 | 2 (1·1) | 128·3 (2·33) | 9·2 |
| 30 - 34 | 1596400 | 59·2 | 4 (1·2) | 236·8 (2·38) | 14·8 |  | 1460900 | 59·2 | 3 (1·65) | 177·6 (3·22) | 12·2 |
| 35 - 39 | 1643800 | 54·3 | 12 (3·59) | 651 (6·54) | 39·6 |  | 1399100 | 54·3 | 4 (2·2) | 217 (3·93) | 15·5 |
| 40 - 44 | 1313900 | 49·3 | 7 (2·1) | 345·2 (3·47) | 26·3 |  | 1094300 | 49·3 | 6 (3·3) | 295·9 (5·36) | 27·0 |
| 45 - 49 | 1090300 | 44·4 | 15 (4·49) | 666·5 (6·7) | 61·1 |  | 961500 | 44·4 | 12 (6·59) | 533·2 (9·66) | 55·5 |
| 50 - 54 | 943700 | 39·6 | 29 (8·68) | 1149·4 (11·55) | 121·8 |  | 895400 | 39·6 | 17 (9·34) | 673·8 (12·21) | 75·3 |
| 55 - 59 | 843400 | 34·9 | 37 (11·08) | 1291·9 (12·98) | 153·2 |  | 780100 | 34·9 | 15 (8·24) | 523·7 (9·49) | 67·1 |
| 60 - 64 | 697700 | 30·3 | 56 (16·77) | 1694·2 (17·02) | 242·8 |  | 659800 | 30·3 | 30 (16·48) | 907·6 (16·45) | 137·6 |
| 65 - 69 | 512200 | 25·7 | 52 (15·57) | 1335·4 (13·42) | 260·7 |  | 494400 | 25·7 | 19 (10·44) | 487·9 (8·84) | 98·7 |
| 70 - 74 | 363900 | 21·3 | 33 (9·88) | 702·5 (7·06) | 193·1 |  | 369000 | 21·3 | 24 (13·19) | 510·9 (9·26) | 138·5 |
| 75 - 79 | 212400 | 17·1 | 33 (9·88) | 564·4 (5·67) | 265·7 |  | 224200 | 17·1 | 19 (10·44) | 325 (5·89) | 144·9 |
| 80 - 84 | 106951 | 13·2 | 23 (6·89) | 304·5 (3·06) | 284·7 |  | 135973 | 13·2 | 12 (6·59) | 158·9 (2·88) | 116·8 |
| 85 - 89 | 52819 | 10·0 | 14 (4·19) | 139·9 (1·41) | 264·8 |  | 63265 | 10·0 | 4 (2·2) | 40 (0·72) | 63·2 |
| 90 - 95 | 18019 | 7·6 | 6 (1·8) | 45·7 (0·46) | 253·7 |  | 18916 | 7·6 | 7 (3·85) | 53·3 (0·97) | 281·9 |
| 95+ | 5411 | 5·9 | 1 (0·3) | 5·9 (0·06) | 109·4 |  | 3646 | 5·9 | 2 (1·1) | 11·8 (0·21) | 324·9 |
| **Total** |  |  | **334 (100)** | **9951·8 (100)** | **56·0** |  |  |  | **182 (100)** | **5517·2 (100)** | **33·5** |
| LE, life expectancy; YLL, years of life lost | | | |  |  |  |  |  |  |  |  |
| LE values based on reference life table from GBD 2019, published by the Institute for Health Metrics and Evaluation (IHME) | | | | | | | | |  |  |  |

**Supplementary Table 2b.** Death counts and years of life lost (YLLs) due to COVID-19 by age and sex in calendar year 2020 in Malaysia; using LE values from the GBD 2019 reference life table (this table assigns the same values to both males and females)

| Age group | Male | | | | |  | Female | | | | |
| --- | --- | --- | --- | --- | --- | --- | --- | --- | --- | --- | --- |
|  | Population | LE | Deaths (%) | YLL (%) | YLL per  100,000 people |  | Population | LE | Deaths (%) | YLL (%) | YLL per  100,000 people |
| <1 | 280700 | 88·9 | 10 (0·06) | 888·7 (0·16) | 316·6 |  | 265440 | 88·9 | 10 (0·08) | 888·7 (0·22) | 334·8 |
| 1 - 4 | 1122800 | 88·0 | 10 (0·06) | 880 (0·15) | 78·4 |  | 1061760 | 88·0 | 12 (0·09) | 1056 (0·26) | 99·5 |
| 5 - 9 | 1391200 | 84·0 | 16 (0·09) | 1344·5 (0·24) | 96·6 |  | 1313400 | 84·0 | 7 (0·05) | 588·2 (0·14) | 44·8 |
| 10 - 14 | 1290300 | 79·0 | 14 (0·08) | 1106·6 (0·19) | 85·8 |  | 1224800 | 79·0 | 10 (0·08) | 790·5 (0·19) | 64·5 |
| 15 - 19 | 1390800 | 74·1 | 29 (0·16) | 2147·9 (0·38) | 154·4 |  | 1308900 | 74·1 | 26 (0·2) | 1925·7 (0·47) | 147·1 |
| 20 - 24 | 1420100 | 69·1 | 125 (0·7) | 8638·4 (1·52) | 608·3 |  | 1338700 | 69·1 | 71 (0·54) | 4906·6 (1·2) | 366·5 |
| 25 - 29 | 1476300 | 64·1 | 268 (1·5) | 17192 (3·03) | 1164·5 |  | 1391100 | 64·1 | 228 (1·72) | 14626 (3·59) | 1051·4 |
| 30 - 34 | 1596400 | 59·2 | 541 (3·04) | 32025·2 (5·64) | 2006·1 |  | 1460900 | 59·2 | 343 (2·59) | 20304·3 (4·98) | 1389·9 |
| 35 - 39 | 1643800 | 54·3 | 799 (4·49) | 43347·8 (7·63) | 2637·1 |  | 1399100 | 54·3 | 596 (4·5) | 32334·6 (7·93) | 2311·1 |
| 40 - 44 | 1313900 | 49·3 | 1090 (6·12) | 53756 (9·46) | 4091·3 |  | 1094300 | 49·3 | 830 (6·26) | 40933·4 (10·04) | 3740·6 |
| 45 - 49 | 1090300 | 44·4 | 1332 (7·48) | 59185·2 (10·41) | 5428·3 |  | 961500 | 44·4 | 1027 (7·75) | 45633 (11·19) | 4746·0 |
| 50 - 54 | 943700 | 39·6 | 1618 (9·09) | 64129 (11·28) | 6795·5 |  | 895400 | 39·6 | 1122 (8·47) | 44470·2 (10·91) | 4966·5 |
| 55 - 59 | 843400 | 34·9 | 1966 (11·04) | 68642·7 (12·08) | 8138·8 |  | 780100 | 34·9 | 1256 (9·48) | 43853·1 (10·75) | 5621·5 |
| 60 - 64 | 697700 | 30·3 | 2217 (12·45) | 67071·9 (11·8) | 9613·3 |  | 659800 | 30·3 | 1493 (11·27) | 45168·4 (11·08) | 6845·8 |
| 65 - 69 | 512200 | 25·7 | 2179 (12·24) | 55958·7 (9·85) | 10925·2 |  | 494400 | 25·7 | 1425 (10·75) | 36595·3 (8·97) | 7402·0 |
| 70 - 74 | 363900 | 21·3 | 1994 (11·2) | 42448·7 (7·47) | 11664·9 |  | 369000 | 21·3 | 1413 (10·66) | 30080·2 (7·38) | 8151·8 |
| 75 - 79 | 212400 | 17·1 | 1491 (8·37) | 25501·3 (4·49) | 12006·3 |  | 224200 | 17·1 | 1075 (8·11) | 18386·3 (4·51) | 8200·8 |
| 80 - 84 | 106951 | 13·2 | 1157 (6·5) | 15317·2 (2·7) | 14321·7 |  | 135973 | 13·2 | 1101 (8·31) | 14575·8 (3·57) | 10719·7 |
| 85 - 89 | 52819 | 10·0 | 661 (3·71) | 6603·5 (1·16) | 12502·2 |  | 63265 | 10·0 | 719 (5·43) | 7182·9 (1·76) | 11353·7 |
| 90 - 95 | 18019 | 7·6 | 224 (1·26) | 1706·4 (0·3) | 9469·9 |  | 18916 | 7·6 | 353 (2·66) | 2689·1 (0·66) | 14215·7 |
| 95+ | 5411 | 5·9 | 67 (0·38) | 396·8 (0·07) | 7333·1 |  | 3646 | 5·9 | 134 (1·01) | 793·6 (0·19) | 21765·5 |
| **Total** |  |  | **17808 (100)** | **568288·5 (100)** | **3197·5** |  |  |  | **13251 (100)** | **407782 (100)** | **2476·7** |
| LE, life expectancy; YLL, years of life lost | | | |  |  |  |  |  |  |  |  |
| LE values based on reference life table from GBD 2019, published by the Institute for Health Metrics and Evaluation (IHME) | | | | | | | | |  |  |  |

**Supplementary Table 2c.** Death counts and years of life lost (YLLs) due to COVID-19 by age and sex in calendar year 2021 in Malaysia; using LE values from the GBD 2019 reference life table (this table assigns the same values to both males and females)

| State / federal territory | Population | Deaths (%) | YLLs (%) | YLL per 100,000 people |
| --- | --- | --- | --- | --- |
| Johor | 3978700 | 3931 (12·26) | 91242·4 (13·34) | 2293·3 |
| Kedah | 2298500 | 2190 (6·83) | 47169·7 (6·9) | 2052·2 |
| Kelantan | 1998200 | 1283 (4) | 23010·1 (3·36) | 1151·5 |
| Melaka | 973200 | 976 (3·04) | 22144·9 (3·24) | 2275·5 |
| Negeri Sembilan | 1172700 | 1340 (4·18) | 26459 (3·87) | 2256·2 |
| Pahang | 1773600 | 811 (2·53) | 17274·7 (2·53) | 974·0 |
| Perak | 2632500 | 1451 (4·53) | 27128 (3·97) | 1030·5 |
| Perlis | 267400 | 138 (0·43) | 2469·6 (0·36) | 923·6 |
| Pulau Pinang | 1825800 | 1774 (5·53) | 32967·8 (4·82) | 1805·7 |
| Sabah | 4111700 | 2839 (8·85) | 53304·8 (7·79) | 1296·4 |
| Sarawak | 2946400 | 1622 (5·06) | 27110·8 (3·96) | 920·1 |
| Selangor | 6815200 | 10072 (31·41) | 236672·8 (34·61) | 3472·7 |
| Terengganu | 1318800 | 760 (2·37) | 15068·1 (2·2) | 1142·6 |
| W.P. Kuala Lumpur | 1925200 | 2703 (8·43) | 57867 (8·46) | 3005·8 |
| W.P. Labuan | 104400 | 151 (0·47) | 3608·8 (0·53) | 3456·7 |
| W.P. Putrajaya | 95900 | 22 (0·07) | 404·4 (0·06) | 421·7 |
| **Total** |  | **32063 (100)** | **683902·9 (100)** | **1997·5** |

**Supplementary Table 3.** Estimated years of life lost (YLL) per 100,000 people in individual states and federal territories, Malaysia
